# Role of Oxidative Stress and Lipid Peroxidation in the Pathophysiology of NAFLD

**DOI:** 10.3390/antiox11112217

**Published:** 2022-11-10

**Authors:** Marta Martín-Fernández, Víctor Arroyo, Carmen Carnicero, Rebeca Sigüenza, Reyes Busta, Natalia Mora, Beatriz Antolín, Eduardo Tamayo, Patricia Aspichueta, Irene Carnicero-Frutos, Hugo Gonzalo-Benito, Rocío Aller

**Affiliations:** 1 BioCritic, Group for Biomedical Research in Critical Care Medicine, 47005 Valladolid, Spain; 2 Department of Medicine, Dermatology and Toxicology, Universidad de Valladolid, 47005 Valladolid, Spain; 3 Research Unit, Hospital Clínico Universitario de Valladolid, 47003 Valladolid, Spain; 4 Centro de Investigación Biomédica en Red de Enfermedades Infecciosas (CIBERINFEC), Instituto de Salud Carlos III, 28029 Madrid, Spain; 5 Institute of Health Sciences of Castile and Leon (IECSCYL), 42002 Soria, Spain; 6 Radiology Unit, Hospital Clínico Universitario de Valladolid, 47003 Valladolid, Spain; 7 Gastroenterology Unit, Hospital Clínico Universitario de Valladolid, 47003 Valladolid, Spain; 8 Anesthesiology and Critical Care Unit, Hospital Clínico Universitario de Valladolid, 47003 Valladolid, Spain; 9 Department of Surgery, Faculty of Medicine, Universidad de Valladolid, 47005 Valladolid, Spain; 10 Department of Physiology, Faculty of Medicine and Nursing, University of the Basque Country UPV/EHU, 48940 Leioa, Spain; 11 Biocruces Bizkaia Health Research Institute, 48903 Barakaldo, Spain

**Keywords:** steatosis, oxidative stress, biomarkers, lipid peroxidation, NAFLD

## Abstract

**Non-alcoholic fatty liver disease** (NAFLD) is characterised by an excess of hepatic fat that can progress to steatohepatitis, fibrosis, cirrhosis and hepatocarcinoma. The imbalance between lipid uptake/lipogenesis and lipid oxidation/secretion in the liver is a major feature of NAFLD. Given the lack of a non-invasive and reliable methods for the diagnosis of non-alcoholic steatohepatitis (NASH), it is important to find serum markers that are capable of discriminating or defining patients with this stage of NASH. Blood samples were obtained from 152 Caucasian subjects with biopsy-proven NAFLD due to persistently elevated liver enzyme levels. Metabolites representative of oxidative stress were assessed. The findings derived from this work revealed that NAFLD patients with a NASH score of ≥ 4 showed significantly higher levels of lipid peroxidation (LPO). Indeed, LPO levels above the optimal operating point (OOP) of 315.39 μM are an independent risk factor for presenting a NASH score of ≥ 4 (OR: 4.71; 95% CI: 1.68–13.19; *p* = 0.003). The area under the curve (AUC = 0.81, 95% CI = 0.73–0.89, *p* < 0.001) shows a good discrimination ability of the model. Therefore, understanding the molecular mechanisms underlying the basal inflammation present in these patients is postulated as a possible source of biomarkers and therapeutic targets in NASH.

## 1. Introduction

Non-alcoholic fatty liver disease (NAFLD) is the main cause of liver disease worldwide, with a prevalence estimated at approximately 25% [1]. NAFLD includes a broad spectrum of liver features, such as steatosis and non-alcoholic steatohepatitis (NASH), which can lead to fibrosis. Fibrosis can progress to cirrhosis and, at worst, hepatocellular carcinoma [2].

The relationship between NAFLD and metabolic syndrome is well known. Metabolic syndrome is characterised by obesity and type 2 diabetes mellitus, as well as dyslipidaemia and hypertension. These features of metabolic syndrome are risk factors that are related to a higher prevalence of NAFLD [3]. Liver biopsy is the gold standard to assess the stage and disease severity; however, there are some limitations, for instance, invasiveness or interobserver variability [4]. For this reason, the need to develop new non-invasive techniques, such as clinical-biochemical non-invasive tests, has arisen [5].

NAFLD is the presence of liver steatosis, defined as the confirmed presence of fat in ≥5% of hepatocytes, given the absence of alcohol intake [6]. Liver enzymes—transaminases—may be elevated, although the disease may exist without that elevation, as seen in advanced fibrosis and cirrhosis [7]. NASH has several features in addition to steatosis, such as hepatocellular damage and inflammatory cell infiltration, which may progress to the development of fibrosis [8]. Fibrosis is defined as the deposition of collagen fibres in the extracellular matrix, which can lead to the development of cirrhosis, thereby increasing the chances of developing hepatocellular carcinoma [9].

Among the pathogenic mechanisms, insulin resistance, inflammation and oxidative stress (OS) should be highlighted as key in disease progression [10].

OS is the homeostatic imbalance between the production of reactive oxygen species (ROS) and their neutralisation by antioxidant defences [11]. The chain of ROS production begins with the formation of the superoxide anion (O2-), which occurs during normal metabolism and leads to the development of the rest of the ROS. The toxic ROS effect is because it can react with biomolecules such as proteins, producing carbonyls; lipids, producing malondialdehyde (MDA) and 4-Hydroxynonenal (HNE); and even DNA, producing the 8-hydroxylation of guanine (8-OHdG), changing their structure and function [12]. Nevertheless, we are protected against the effect of ROS by the antioxidant defences that can be classified into enzymatic and non-enzymatic forms. On the one hand, the enzymes are superoxide dismutase (SOD), which dismutase the superoxide anion into hydrogen peroxide, and catalase (CAT), which neutralises hydrogen peroxide in water. On the other hand, non-enzymatic forms cover a wide range of molecules, such as glutathione, although the vast majority occur via food (tocophenols, isoflavones, vitamins, polyphenols, etc). The total concentration of these non-enzymatic molecules is called the antioxidant capacity [12].

The free radicals that induce OS are crucial to the progression of NAFLD because this can lead to lipid peroxidation (LPO) failures on the mitochondrial oxidation of fatty acids, aside from cytokine release, which induces inflammation. Therefore, OS can lead to hepatocellular damage [13].

Lipid peroxidation starts when a hydrogen atom is abstracted from an unsaturated fatty acid by a radical—ROS [14]. This is the beginning of a destructive chain reaction that results in the disruption of membranes and the production of reactive metabolites that may cause cellular disfunction. Lipid peroxidation and its products activate hepatic stellar cells and upregulate the expression of proinflammatory cytokines [15]; they may also activate cell necrosis and the apoptotic Fas-ligand pathway, which has been implicated in the development of fibrosis [16].

Studies evaluating OS in NAFLD are scarce. In this regard, here, we aimed to perform a complete oxidative stress profile evaluation regarding antioxidant enzymes, total antioxidant capacity and oxidative cell damage, specifically lipid peroxidation, in NAFLD, which could be useful in the search for diagnostic and therapeutic targets.

## 2. Materials and Methods

### 2.1. Subjects

The study group consisted of 152 Caucasian subjects with biopsy-proven NAFLD (body mass index: 33.3 ± 7.2 Kg/m^2^) due to persistently elevated liver enzyme levels. The exclusion criteria applied included significant alcohol consumption (>30 g/day in men and >20 g/day in women), hepatitis B, hepatitis C, cytomegalovirus or Epstein–Barr virus infection, positive non-organ-specific autoantibodies, type 1 diabetes mellitus, antihypertensive drug or statin therapy, and primary metabolic diseases (iron and copper storage diseases and alpha 1-antitrypsin deficiency). Patients were split into two groups according to their NASH score, considering a cut-off value of 4 points [17]. All participants signed the corresponding informed consent form. The study was conducted according to the guidelines of the Declaration of Helsinki. The Ethics Committee of Hospital Clínico Universitario de Valladolid (protocol code PI 20-1818) approved all procedures involving patients, and patient data were coded in order to guarantee anonymity.

The diagnosis of NAFLD was established from the liver biopsy. Basal glucose, C-reactive protein (CRP), insulin, homeostasis model assessment-insulin resistance (HOMA-IR), total cholesterol, LDL-cholesterol, HDL-cholesterol and triglycerides were all determined. 

### 2.2. Liver Histology

The diagnosis of NAFLD was confirmed by a percutaneous liver biopsy using a Menghini-type biopsy needle in all patients. Liver samples were sectioned and stained with haematoxylin-eosin and the Masson trichrome stain. NAFLD was histologically defined by the presence of a minimum of 5% steatosis in the liver biopsy. The degree of steatosis in turn was scored as 1 (5–33%), 2 (34–66%) or 3 (>66%). Fibrosis was scored as 0 (no fibrosis), 1 (peri-sinusoidal or periportal fibrosis), 2 (peri-sinusoidal and portal/periportal fibrosis), 3 (bridging fibrosis) or 4 (cirrhosis). Lobular inflammation was scored as 0 (no inflammation), 1 (<2 foci per 200× field), 2 (2–4 foci per 200× field) or 3 (>4 foci per 200× field). Ballooning was scored as 0 (no ballooned cells), 1 (a few ballooned cells) or 2 (many cells/prominent ballooned cells). A case presenting with at least grade 1 of each of the three abovementioned features (steatosis, ballooning and lobular inflammation) was classified as NASH [18].

In order to minimise inter-observer variability, the liver biopsy specimens were interpreted by the same pathologist using the SAF (Steatosis, Activity, Fibrosis) score, which assesses the grade of steatosis (S) (from S0 to S3), the grade of activity (A) (from A0 to A4 by adding grades of ballooning and lobular inflammation, both from 0 to 2) and the stage of fibrosis (F) (from F0 to F4) [19]. In addition, biopsies were digitised and confirmed by external pathologists.

### 2.3. Biochemical Parameters

Blood samples were collected in Na-EDTA tubes from patients after 12 h of fasting. Insulin was measured by radioimmunoassay (RIA Diagnostic Corporation, Los Angeles, CA, USA) with a sensitivity of 0.5 mIU/L (normal range 0.5–30 mIU/L) [20]. The homeostasis model assessment–insulin resistance (HOMA-IR) was calculated using the following formula: (fasting insulin x fasting glucose concentrations/22.5. The LDL cholesterol levels were calculated using the Friedewald formula [21].

### 2.4. Oxidative Stress Determinations

The determination of levels of antioxidant enzyme activity (superoxide dismutase and catalase) and total antioxidant capacity using the ABTS and FRAP methods was performed. In addition, DNA oxidised guanosine species (8-OHdG) and lipid peroxidation (MDA + HNE) were measured as markers of oxidative cell damage. All of these determinations were performed in plasma samples from the patients as described by Martín-Fernández et al. [22] and Gonzalo et al. [23].

Superoxide dismutase activity was assessed by using the Superoxide Dismutase (SOD) Colorimetric Activity Kit (ref. K028-H1 from Arbor Assays, MI, USA) following the manufacturer’s recommendations. The colour intensity at 440 nm was used to determine the SOD activity.

Catalase (CAT) activity was determined by using the Catalase (CAT) Activity Assay Kit (ref. E-BC-K031-M from Elabscience, TX, USA) following the manufacturer’s recommendations. CAT activity was calculated by production of the yellowish complex at 405 nm.

The antioxidant capacity of samples was evaluated by two methods: 

FRAP (Ferric Reducing Antioxidant Power): This technique is based on the capacity of the sample to allow iron reduction, which is performed as described by Benzie and Strain. Results were quantified by absorbance at 595 nm using a standard curve of known Trolox 6-hydroxy-2,5,7,8-tetramethychroman-2-carboxylic acid concentrations (Aldrich Chemical Co., Gillingham, Dorset, UK), following our lab protocol.

ABTS (2,2-azino-bis (3-ethylbenzthioziozline-6-sulfonic acid): This assay is based on an antioxidant capacity estimation by the performance of a colorimetric test using the cationic radical (ABTS). The ABTS (ABTS, Sigma-Aldrich, St. Louis, MO, USA) was dissolved with 2.45 mM potassium persulphate to make a final concentration of 7 mM. The ABTS radical cation was produced by reacting ABTS stock solution with 2.45 mM potassium persulfate and leaving it to stand in the dark at room temperature overnight. Trolox (6-hydroxy-2,5,7,8-tetramethychroman-2-carboxylic acid; Aldrich Chemical Co., Gillingham, Dorset, UK) was used to construct a standard curve. After adding ABTS solution (1:100), absorbance was measured at 405 nm between 0 and 6 min. Results were expressed as the Trolox equivalent antioxidant capacity (TEAC) of each sample. This assay was performed by following our lab protocol.

The DNA oxidised guanosine species, 8-hydroxy-2’-deoxyguanosine (8-OHdG), was determined at a wavelength of 450 nm, following the manufacturer’s recommendations and using The DetectX^®^ DNA Damage Immunoassay Kit (Arbor Assays, Ann Arbor, MI, USA). 

Lipid peroxidation (LPO) products were analysed using the Bioquochem commercial kit ref KB03002 (BQCell™ MTT, Bioquochem, Oviedo, Spain) following the manufacturer’s recommendations. Malondialdehyde (MDA) and 4-Hydroxynonenal (HNE) concentrations were measured from 580–620 nm as an index of lipid peroxidation.

### 2.5. Statistical Analysis

The differences between groups were assessed by using the Mann–Whitney U test. The differences in the oxidative stress levels of molecules between groups were assessed using the Mann–Whitney U test. The optimal operating point (OOP) of lipid peroxidation (LPO) was calculated as the value for which the point on the curve had the minimum distance to the upper left corner (where sensitivity = 1 and specificity = 1). According to Pythagoras’ theorem, this distance is: Optimal Operating Point (OOP) = √(1−sensitivity)2 + (1−specificity). A Wald backward stepwise multivariable logistic regression analysis was performed to evaluate the association between LPO and NASH score of ≥ 4 risk. Variables showing a *p*-value of < 0.1 with no collinearity in the univariate regression analysis were included in the multivariate analysis as adjusting variables. The discrimination ability of the model was evaluated by performing a Receiving Operating Characteristic (ROC) curve analysis. We considered 2-sided *p*-values of < 0.05 to indicate statistical significance. All data were analysed using the IBM SPSS 22.0 software (SPSS, Chicago, IL, USA).

## 3. Results

### 3.1. Clinical Characteristics

A total of 152 patients were registered in the study and divided into two groups: NASH score < 4 (n = 82) and NASH score ≥ 4 (n = 70), which represents 46% of the cohort. Although NASH CRN does not exactly define NASH by using the NASH score [24], we determined NASH according to the NASH score of ≥ 4 (with at least 1 point each in inflammation and ballooning) [25]. This threshold was based on the inclusion criteria of most of the Food and Drug Administration (FDA)-approved clinical trials to develop NAFLD therapeutic drugs [26]. Clinical characteristics of patients are shown in Table 1. In terms of age, there were no differences between groups, as was the case for body mass index, with all patients being overweight. Male gender was more frequent in the group with a NASH score of < 4, while type 2 diabetes mellitus was more common in the group presenting a NASH score of ≥ 4. Regarding the histological characteristics, as expected, patients with a NASH score of ≥ 4 presented the presence of moderate/severe steatosis, NASH (inflammation and ballooning) and advanced fibrosis more frequently. This group of patients presenting a NASH score of ≥ 4 showed higher blood pressures and, as expected, higher Fibroscan values, higher levels of AST and ALT, and higher scores on the non-invasive hepatic steatosis index (HIS), non-alcoholic fatty liver disease fibrosis score (NFS) index and AST to platelet ratio index (APRI). These patients also presented higher levels of HOMA-IR, glycaemia and systolic blood pressure, while creatinine and albumin levels were lower in comparison with the other group.

### 3.2. Oxidative Stress Levels in NAFLD

The comparison of NAFLD patients presenting a NASH score of ≥ 4 with those presenting a NASH score of < 4 revealed that, of all of the oxidative stress molecules analysed, only LPO levels (MDA+HNE) were statistically different between groups. No statistically significant differences were, therefore, identified for antioxidant enzymes (SOD, Catalase), total antioxidant capacity (ABTS, FRAP) and the DNA damage marker 8-OHdG (Table 2 and Figure S1). This same analysis was performed by stratifying patients according to the presence of diabetes and the presence of obesity (body mass index > 30). In the case of stratifying patients by the presence/absence of diabetes, none of the oxidative stress molecules were significant in the comparison of patients with a NASH score of ≥ 4 and those with a NASH score of < 4. On the other hand, when stratifying patients according to the presence/absence of obesity, lipid peroxidation showed significantly higher levels in patients with a NASH score of ≥ 4, as previously reported, and the presence of obesity. Therefore, the results observed in Table 2 and Figure S1 could be especially attributable to this population of patients with obesity in our cohort (data not shown).

### 3.3. Evaluation of the Risk of NASH Score Depending on LPO Levels

**Figure 1:**
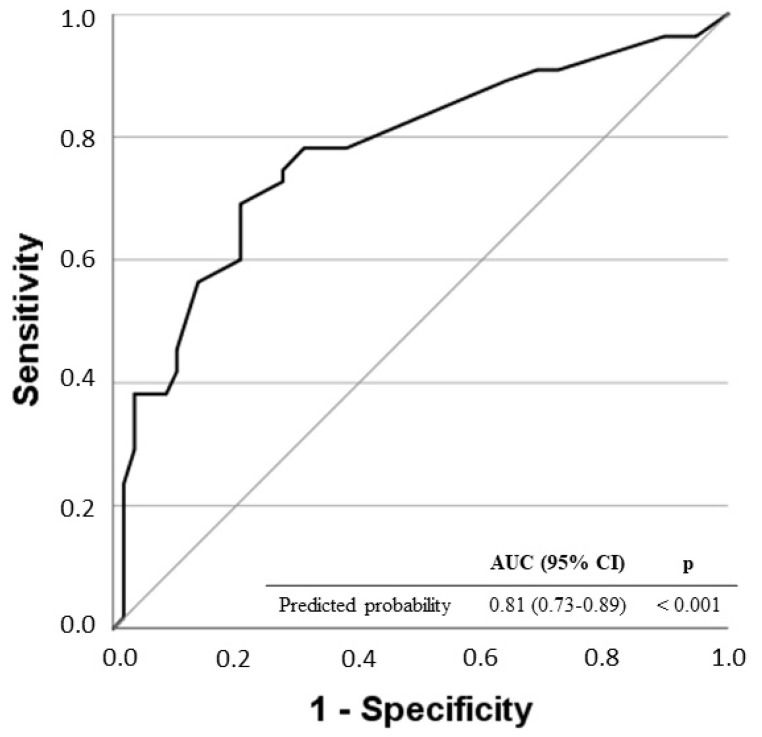
Receiver operating characteristic (ROC) curves for the predicted probability of the multivariate logistic regression model for a NASH score of ≥ 4.

Multivariate regression analysis identified LPO levels over the OOP (LPO > 315.39 μM) as an independent risk factor of the NASH score of ≥ 4 in NAFLD patients [OR: 4.71; 95% CI: 1.68–13.19; *p* = 0.003] (Table 3). The ROC curve of predicted probability for the multivariate logistic regression model is shown in Figure 1. The area under the curve (AUC = 0.81, 95% CI = 0.73–0.89, *p* < 0.001) shows the good discrimination ability of the model.

## 4. Discussion

This study evaluated, for the very first time, an extensive and representative oxidative stress profile regarding antioxidant enzymes (SOD and Catalase), total antioxidant capacity (ABTS and FRAP) and oxidative cell damage (8-OHdG and LPO) in patients with NAFLD. The findings derived from this work revealed that NAFLD patients with a NASH score of ≥ 4 showed significantly higher levels of lipid peroxidation. Indeed, LPO levels above 315.39 μM are an independent risk factor for presenting a NASH score of ≥ 4. 

NAFLD has been considered a complex disease, which is also frequently associated with other pathologies such as diabetes and cardiovascular and chronic kidney diseases [26]. Multiple studies have revealed that NAFLD is a hepatic manifestation of metabolic syndrome, characterised by pathological changes in carbohydrate and lipid metabolism [27,28].

In NAFLD, the increased lipid storage into the hepatocytes induces the production of high levels of reactive oxygen species (ROS), which leads to LPO [26]. LPO is a biological free radical chain reaction. The oxidation of unsaturated FAs or other lipids generates peroxides of these compounds. Increased LPO has been documented in animal models [29]. Abnormal peroxidation occurs both in the liver cell and in circulating lipids, leading to lipotoxicity with metabolic dysfunctions [30,31].

Oxidative stress has been postulated as a central process contributing to liver damage, stimulating the transition from simple steatosis to NASH [32]. Mitochondrial dysfunction in liver tissue during NAFLD has been reported to affect the liver lipid equilibrium, promote ROS production and affect LPO and cytokine release, ultimately leading to cell death [33]. As a consequence of the damage to mitochondrial function in NAFLD, the mitochondria become more susceptible to mitochondrial permeability transition (MPT), subsequently leading to cell death when undergoing ischemia and reperfusion injury [34]. Mitochondrial membrane phospholipids are oxidised, reducing fluidity and hampering the entry of glutathione (GSH) into the mitochondria, provoking an imbalance between antioxidants and ROS. This induces oxidative stress, which leads to a reduction in mitochondrial ATP synthesis [35]. Consequently, NAFLD patients have diminished ATP reserves, leaving cells more susceptible to necrosis and triggering an inflammatory response when the liver is injured by ischemia and hypoxia [34].

ROS bind to polyunsaturated fatty acids (PUFA) forming lipid peroxides. Unstable lipid peroxides are readily decomposed into active 4-hydroxy-2-nonenal (4-HNE) and malondialdehyde (MDA), resulting in cell damage [36,37]. Lipids are the most vulnerable to ROS attack in fatty liver ischemia and reperfusion injury. However, FA accumulation and extensive lipid peroxidation are contained within NAFLD itself, making fatty liver ischemia and reperfusion injury more severe [38]. In the pathogenesis of NAFLD, ROS also oxidises proteins, particularly antioxidant enzymes, and the antioxidant capacity is impaired after oxidation.

In the studies of Ampuero et al., a relatively high proportion of patients did not show findings related to definite NASH (in particular, half of the cirrhotic patients and one third of those with advanced fibrosis could not be diagnosed with NASH) [17]. Furthermore, a recent Italian study [39] showed that up to 33% of patients with significant NAFLD-related fibrosis did not show NASH (up to 10% had no inflammation). Therefore, the results of our work show that by determining LPO markers, we can identify those patients avoiding the risks and variability of biopsy with high diagnostic accuracy, which could also be useful as a therapeutic tool.

At present, more than 100 indexes have been developed to assess liver injury or to predict cardiovascular, neoplastic or progression-related episodes of cirrhosis, hepatocarcinoma or episodes of decompensation of cirrhosis. In general, however, these are combinations of similar anthropometric and biochemical parameters, although more progress has been made in markers of fibrosis than of NASH. The incorporation of methods obtained from transcriptomics, genomics, metabolomics or proteomics studies does not seem to significantly improve performance [40]. OWLiver (metabolomic profiling) [41] and ELF (European Liver Fibrosis) [42] have been recommended as second-line methods. However, there is a lack of non-invasive indices based on the pathophysiological mechanisms of the disease such as those derived from oxidative stress and LPO; as proposed, that will allow a better diagnostic approach both in real life and in the context of clinical trials. 

Our study has some limitations to be addressed. First, oxidative stress biomarkers were compared only at a single time point. Second, it was conducted in a single centre and should be evaluated in a multicentre fashion design to validate the potential role of lipid peroxidation in the diagnosis of NASH.

## 5. Conclusions

In summary, our findings show that higher lipid peroxidation levels are independently associated with a greater risk of presenting a NASH score of ≥ 4. Therefore, these results reveal that lipid peroxidation levels are a promising and valuable diagnostic tool for detecting NASH patients suffering from NAFLD. This study will pave the way for further clinical studies to understand the correlation between lipid peroxidation and basal inflammation in this population, which will guide the development of new therapies and the discovery of new biomarkers.

## Figures and Tables

**Table 1:** Clinical characteristics of NAFLD patients split by a NASH score of 4. Continuous variables are represented as [median, (interquartile range, IQR)]; categorical variables are represented as [%, (n)]. HOMA-IR, Homeostatic Model Assessment for Insulin Resistance; AST, Aspartate Aminotransferase; ALT, Alanine Aminotransferase; GGT, Gamma-glutamyl transferase; FLI, Fatty Liver Index; HSI, Hepatic Steatosis Index; NFS, Non-Alcoholic Fatty Liver Disease fibrosis score; FIB4, Fibrosis-4; HFS, Hepamet Fibrosis Score; APRI, AST to Platelet Ratio Index.

	NASH Score < 4 (N = 82)	NASH Score ≥ 4 (N = 70)	*p*
** -Demographic **			
Age [years, median (IQR)]	49 (18.50)	51.50 (23)	0.93
Male [%(n)]	69.5 (57)	48.6 (34)	** 0.009 **
** -Comorbidities, [%(n)] **			
Diabetes	11.3 (7)	28.6 (18)	** 0.016 **
Hypercholesterolemia	49.4 (39)	47.8 (33)	0.85
Hypertriglyceridemia	34.2 (26)	38.5 (25)	0.60
Obesity	61.7 (50)	68.6 (48)	0.38
** -Liver biopsy, [%(n)] **			
Mild Steatosis (<33%)	73.2 (60)	12.9 (9)	** <0.001 **
Moderate/Severe Steatosis (≥33%)	26.8 (22)	87.1 (61)	** <0.001 **
NASH (Inflammation + Ballooning)	20.7 (17)	88.6 (62)	** <0.001 **
Advanced/Severe Fibrosis (≥F3)	9.8 (8)	25.7 (18)	** 0.009 **
Cirrhosis (F4)	3.7 (3)	11.4 (8)	0.06
** -Laboratory measurements, [median (IQR)] **			
Height (m)	1.67 (0.13)	1.62 (0.17)	0.09
Weight (kg)	86.3 (21.95)	87.6 (29.35)	0.88
Body Index Mass (BMI)	31.05 (36.91)	32.30 (16.02)	0.24
Waist (cm)	104 (19.50)	105 (20.65)	0.67
Hip (cm)	107 (16.38)	108.25 (15.65)	0.90
Systolic Blood Pressure (mmHg)	120 (35)	140 (28.75)	** <0.001 **
Diastolic Blood Pressure (mmHg)	75 (30)	90 (16)	** <0.001 **
Fibroscan * (KPa)	8.1 (6.4)	10.35 (7.55)	** 0.033 **
AST (U/L)	30 (17.50)	41 (27)	** <0.001 **
ALT (U/L)	52.50 (29)	65 (51)	** 0.002 **
GGT (U/L)	82.50 (97.25)	68 (73)	0.45
HOMA-IR score	3.42 (4.44)	5.37 (5.08)	** <0.001 **
Total Cholesterol (mg/dL)	183 (50)	192 (59.25)	0.58
HDL Cholesterol (mg/dL)	43.50 (12.15)	46.70 (12.80)	0.59
LDL Cholesterol (mg/dL)	106.60 (31.38)	116 (51)	0.49
Triglycerides (mg/dL)	134 (96.75)	133 (99)	0.62
Glycaemia (mg/dL)	103 (25)	109 (32.50)	** 0.011 **
Creatinine (mg/dL)	0.90 (0.28)	0.80 (0.28)	** 0.003 **
Uric acid (mg/dL)	5.90 (1.50)	5.80 (2.03)	0.55
Total bilirubin (mg/dL)	0.62 (0.50)	0.60 (0.40)	0.37
Alkaline Phosphatase (ALP)	72 (32.25)	80 (32)	** 0.039 **
Leukocytes (cells/mL)	6760 (5750)	5745 (5170)	0.28
Platelet (cells/mL)	254,000 (95,000)	242,000 (122,250)	0.35
Ferritin (ng/mL)	186 (164.20)	174 (180.20)	0.93
Albumin (g/dL)	4.60 (0.70)	4.40 (0.70)	** 0.005 **
FLI score	85.26 (84.03)	81.37 (73.23)	0.68
HSI score	42.07 (33.22)	45.80 (28.73)	** 0.033 **
NFS	−3.59 (3.46)	−2.05 (2.92)	** 0.005 **
FIB4 score	0.85 (0.64)	1.06 (1.15)	0.07
HFS score	0.019 (0.06)	0.12 (0.36)	** <0.001 **
APRI score	−3.48 (6.72)	0.33 (10.55)	** 0.001 **

**Table 2:** Oxidative stress molecules’ levels in NAFLD patients split by a NASH score of 4. Data are represented as median and interquartile range (IQR).

	NASH Score < 4	NASH Score ≥ 4	*p*
**SOD (U/mL)**	0.14 (0.06)	0.13 (0.06)	0.518
**Catalase (U/μL)**	42.40 (45.84)	34.02 (39.65)	0.384
**FRAP (μM)**	409.64 (110.65)	392.71 (130.14)	0.826
**ABTS (μM)**	548.43 (477.99)	560.09 (556.05)	0.496
**8-OHdG (pg/mL)**	17710 (24974)	19395 (33234)	0.370
**MDA + HNE (μM)**	120.48 (280.61)	230.14 (355.89)	**0.024**

**Table 3:** Automatic multivariate logistic regression analysis to evaluate the independent association of LPO levels and risk of a NASH score of ≥ 4.

	OR	95% CI	*p*
HSI	1.05	1.02–1.09	<0.001
FIB4	1.68	1.05–2.68	0.030
APRI	1.08	1.02–1.13	0.005
**LPO > 315.39 μM**	4.71	1.68–13.19	0.003

## Data Availability

The datasets during and/or analysed during the current study available from the corresponding author upon reasonable request.

## Supplementary Materials

The following supporting information can be downloaded at: https://www.mdpi.com/article/10.3390/antiox11112217/s1, Figure S1: Box plots showing oxidative stress molecules’ levels across groups.
