# Supplementary figures and images for: Role of Oxidative Stress and Lipid Peroxidation in the Pathophysiology of NAFLD
Source: Antioxidants (Basel). 2022 Nov 10;11(11):2217. doi: 10.3390/antiox11112217 (PMC9686676; doi:10.3390/antiox11112217)

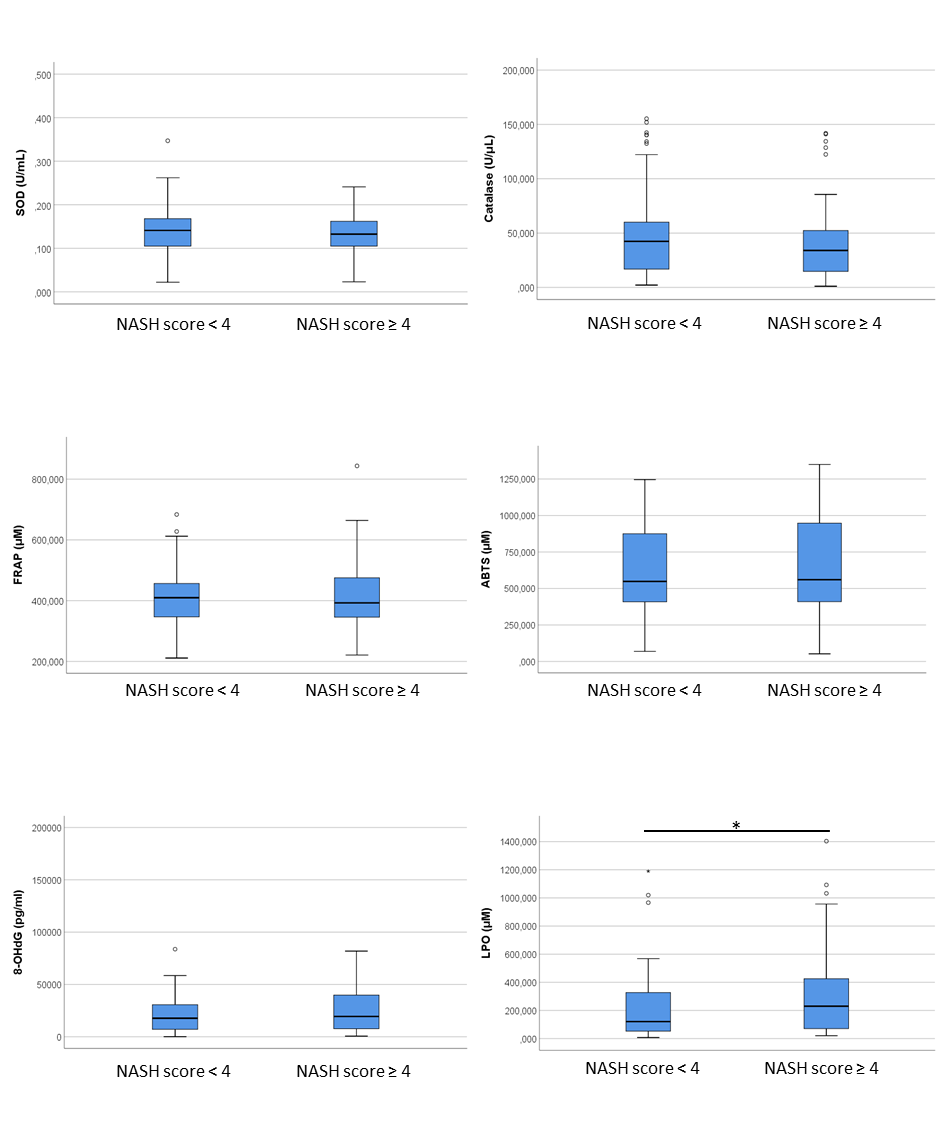

Supplement: Supplementary file 1 [file antioxidants-11-02217-s001.zip › antioxidants-1993675-supplementary.tif]
